# Chronically ill patients’ self-management abilities to maintain overall well-being: what is needed to take the next step in the primary care setting?

**DOI:** 10.1186/s12875-015-0340-8

**Published:** 2015-09-15

**Authors:** Jane Murray Cramm, Anna Petra Nieboer

**Affiliations:** Institute of Health Policy & Management (iBMG), Erasmus University, Rotterdam, The Netherlands

**Keywords:** Shared decision making, Patient-centered care, Patient preferences, Quality of care, Productive patient-professional interaction, Self-management

## Abstract

**Background:**

Although widespread problems in patient–professional interaction and insufficient support of patients’ self-management abilities have been recognized, research investigating the relationships among care quality, productive interaction, and self-management abilities to maintain overall well-being is lacking. Furthermore, studies have revealed differences in these characteristics among certain groups (e.g., less-educated and older patients). This longitudinal study thus aimed to identify relationships among background characteristics, quality of care, productivity of patient–professional interaction, and self-management abilities to maintain overall well-being in chronically ill patients participating in 18 Dutch disease management programs.

**Methods:**

This longitudinal study included patients participating in 18 Dutch disease management programs. Surveys were administered in 2011 (T1; *n* = 2191 (out of 4693), 47 % response rate) and 2012 (T2: *n* = 1722 (out of 4350), 40 % response rate). A total of 1279 patients completed questionnaires at both timepoints (T1 and T2) (27 % response rate). Self-management abilities to maintain well-being were measured using the short (18-item) version of the Self-Management Ability Scale (SMAS-S), patients’ perceptions of the productivity of interactions with health care professionals were assessed with the relational coordination instrument and the short (11-item) version of the Patient Assessment of Chronic Illness Care (PACIC-S) was used to assess patients’ perceptions of the quality of chronic care delivery.

**Results:**

Perceived and objective quality of care and the productivity of patient–professional interaction were found to be related to patients’ self-management abilities to maintain overall well-being. These abilities were related negatively to and significantly predicted by low educational level, single status, and older age, despite the mediating role of productive interaction in their relationship with patients’ perceptions of care quality.

**Conclusions:**

These findings suggest that patient–professional interaction is not yet sufficiently productive to successfully protect against the deterioration of self-management abilities in some groups of chronically ill patients, although such interaction and high-quality care are important factors in such protection. Improvement of the quality of chronic care delivery should thus always be accompanied by investment in high-quality communication and patient–professional relationships.

**Electronic supplementary material:**

The online version of this article (doi:10.1186/s12875-015-0340-8) contains supplementary material, which is available to authorized users.

## Background

Support of chronically ill patients’ self-management abilities has been identified as a key component of effective chronic care delivery [1–4]. Chronically ill patients require self-management support beyond traditional acute care delivery, including the strengthening of problem-solving skills and improvement of self-efficacy and the ability to deal with real-life situations that matter to them [5]. Such care incorporates the whole person, not merely the object of disease [6], and should thus aim to strengthen abilities to maintain overall well-being [7, 8]. Such abilities have been found to be closely related to physical health and depressive symptoms among various patient populations, such as those with cardiovascular diseases (CVDs), diabetes, or chronic obstructive pulmonary disease (COPD) [7]. Self-management abilities also seem to mediate the relationships between social, cognitive, and physical functioning and well-being among patients with these chronic conditions [9] and among older adults after hospitalization [10]. Functional decline and subsequent deterioration of well-being will thus occur only when self-management abilities are poor.

Based on the self-management of well-being theory, Steverink, Lindenberg, and Slaets [11] identified self-management abilities, such as self-efficacy (a person’s belief in his/her ability to accomplish a certain goal), the ability to take initiative (self-motivation or playing an instrumental role in the maintenance of one’s well-being), and investment behavior (e.g., investing in resources to maintain good health and social relationships), that allow individuals to achieve long-term benefits and maintain well-being. These abilities enhance vulnerable elderly or chronically ill patients’ reserve capacities to realize and sustain overall well-being, beyond merely dealing with a chronic condition. Living with a chronic illness can be burdensome, and having to deal with pain, treatment adherence, and low energy level may increase the difficulty of taking the initiative to keep oneself busy, ensuring that one has enough interests on a regular basis (e.g., a hobby) to keep active, and maintaining positive thinking. Hence, chronically ill patients’ self-management abilities to maintain overall well-being may weaken as a consequence of living with a chronic condition and possible deterioration of functional capacities [8].

Although we lack knowledge about how best to improve the self-management abilities of these patients in the primary care setting [12–14], we do know that effective and high-quality chronic care, including self-management interventions that actively involve chronically ill patients and improve their well-being, is needed [13]. The multiple and often complex needs of these patients require an emphasis on coordinated, comprehensive care along the continuum of disease and across health-care delivery systems [15]. Current care delivery, however, remains focused primarily on acute care and short-term goals that emphasize the management of disease complications, typically without self-management support [3]. High-quality chronic care delivery calls for a comprehensive disease management approach with multidisciplinary teams (e.g., nurses, therapists, social workers, pharmacists, and dieticians) to support patients over time, including the offering of self-management support services [1, 16], and is thus expected to enhance chronically ill patients’ self-management abilities to maintain overall well-being. Disease management programs aiming to improve the quality of chronic care through comprehensive system changes are often based on the chronic care model (CCM) [1–3], which incorporates the following six interrelated components of health care systems: (i) self-management support, (ii) delivery system design, (iii) decision support, (iv) clinical information systems, (v) health care organization, and (vi) community linkages. The goal of this model is to transform chronic disease care from acute and reactive to proactive, planned, and population based [3]. Primary care practices that employ the CCM (i) support the self-management abilities of chronically ill patients through lifestyle programs, skill building, educational materials, and group classes; (ii) redesign the way that care is delivered to these patients, which requires well-trained professionals that ensure successful self-management, coordinate preventive care, and screen for common comorbidities; (iii) make use of decision support resources, such as evidence-based practice guidelines (e.g., care standards and clinical guidelines), which are critical for the optimal management of any chronic illness in order to provide high-quality care; and (iv) implement information systems to improve communication and coordination among participating professionals and provide timely reminders and feedback to these professionals. These four dimensions of primary care practices function within the wider context of (v) a health care system that provides incentives to improve the quality of chronic care delivery (e.g., through bundled payments for disease management) and (vi) a community that supports such delivery (e.g., close collaboration with hospitals *via* transmural care protocols and/or pathways and the intensification of relationships with neighborhood exercise programs).

High-quality chronic care delivery based on the CCM supports productive interaction between patients and professionals, leading to better patient outcomes [2, 3]. Such interaction has been identified as important for the effective support of patients’ self-management abilities [1–4], for example, by identifying problems from the patient’s perspective and tailoring knowledge and education to each patient’s needs [5]. Gittell [16, 17] identified this concept as “relational coproduction,” which refers to the coproduction of care delivery through combined equal contributions of patients and their health care providers. Such interactions are based on high levels of shared goals, shared knowledge, and mutual respect that together foster attentiveness to the situation and to one another [17]. Productive patient–professional interaction involves the presentation of options and provision of high-quality information enabling patients to participate more actively in care, by considering the best available evidence and patients’ preferences, as well as understanding patients as persons [18–21]. Patients’ preferences are elicited and explored, and professionals ask questions to help patients construct these preferences [22] and make decisions together. The provision of high-quality information is the first step. Research has shown that patients who are informed about their options have a greater desire to be involved in health decisions than do uninformed patients [23]. Especially with regard to self-management abilities for the maintenance of overall well-being, the alignment of care delivery with the patient as a whole, beyond the chronic condition, is important [8]. Too often, however, care is characterized by interactions between uninformed chronically ill patients and unprepared professionals, resulting in feelings of frustration [3] and a missed opportunity to improve self-management abilities.

Although widespread problems in patient–professional interaction and insufficient support of patients’ self-management abilities have been recognized [24], research investigating the relationships among care quality, productive interaction, and self-management abilities to maintain overall well-being is lacking. Furthermore, studies have shown that self-management abilities are related to chronically ill patients’ socioeconomic and educational levels, marital status, gender, and age [7, 25, 26]. Active self-management of a chronic condition may, for example, be easier for younger, more-educated patients belonging to a higher social class than for those who have lower social status and are older and/or less educated (and thus lack necessary resources) [25, 26]. As the quality of chronic care delivery has also been associated with chronically ill patients’ educational level and age [27, 28], these differences may also apply to patient–professional interaction. Productive interaction may be more difficult among certain patient populations. Thus, this study aimed to identify relationships among chronically ill patients’ background characteristics, quality of care, patient–professional interaction, and self-management abilities to maintain overall well-being.

The dependent variable, self-management abilities to maintain overall well-being, is conceived of as being influenced by patient–professional interaction, with more productive interaction assumed to result in a greater increase (or smaller decrease) in self-management abilities. This improvement (or reduced deterioration) in abilities also depends on the quality of care, as high-quality care facilitates productive patient–professional interaction. In turn, patients’ background characteristics affect care quality and self-management abilities, with stable and dynamic characteristics determining their ability to maintain well-being [29, 30] and old age, single status, and low educational level expected to result in the delivery of lower-quality chronic care, occurrence of fewer productive interactions, and deterioration of self-management abilities.

## Methods

This longitudinal study included patients participating in 18 Dutch disease management programs implementing care based on the CCM, characterized as collaborations between care sectors [e.g., between general practitioners (GPs) and hospitals] or within primary care settings (e.g., among pharmacists, physiotherapists, dieticians, and social workers). In 2008 a national program on “disease management of chronic diseases” was carried out by ZonMw (Netherlands Organisation for Health Research and Development) and commissioned by the Dutch Ministry of Health. Funding was provided for practices planning a redesigning of primary care according to the CCM. Requirements of the national program were that the practices had to have some experience with the delivery of chronic care and were equipped to implement all systems needed for the delivery of sufficient chronic care, which resulted in the inclusion of 22 disease management programs (out of a total of 38 programs who applied for funding). We evaluated these 22 disease management programs that aimed to enhance knowledge on disease-management experience in chronic disease care and stimulate implementation of successful programs [31]. Four disease management programs were excluded due to differences in the timing of questionnaire distribution (*n* = 1) and questionnaire content [to address specific mental health conditions (psychotic disorders, depression, and eating disorders); *n* = 3] which led to a total of 18 disease management programs in the current study. The disease management programs included in the study targeted patients with CVDs (*n* = 9), COPD (*n* = 4), heart failure (*n* = 1), comorbidity (*n* = 1), and diabetes (*n* = 3). The ethics committee of the Erasmus University Medical Center of Rotterdam approved the study and all participants provided informed consent.

In 2011 (T1), we sent questionnaires to all 4702 patients who had participated in the 18 disease management programs for about 1 year; 2346 respondents completed the questionnaire (50 % response rate). Most important reasons for drop out of patients at 2011 and 2012 were: filling in the questionnaire is too time consuming, not interested to participate, patients do not feel they are chronically ill, not able to understand all questions (poor level of the Dutch language), patient had moved, or the address was not correct. In only a few cases patients were too ill (due to a stroke, dementia) or died during the past year. One year later (2012; T2), we sent questionnaires to 4137 patients still participating in the disease management programs; 1851 respondents completed the questionnaire at this timepoint (45 % response rate). All patients enrolled in the disease management programs at the beginning of the evaluation were invited to fill in the questionnaire at T2, not just the patients who responded to the T1 questionnaire. A total of 1279 patients completed questionnaires at both timepoints (T1 and T2). See appendix for a full overview of the response rates at both timepoints in each disease management program.

### Measures

Self-management abilities to maintain overall well-being were measured at T1 and T2 using the short (18-item) version of the Self-Management Ability Scale (SMAS-S) [32, 33]. This instrument assesses a broad repertoire of self-management abilities. The initiative taking, investing, self-efficacy, variety, and multifunctionality subscales are related to the physical and social dimensions of well-being, and the subscale measuring the ability to have a positive frame of mind is considered to be a more general cognitive frame. Average scores range from 1 to 6, with higher scores indicating better self-management abilities. The Cronbach’s alpha value of the SMAS-S at T1 and T2 was 0.92, indicating excellent reliability.

We assessed patients’ perceptions of the productivity of interactions with health care professionals (GPs, practice nurses, dieticians, physical therapists, medical specialists, and nurses) involved in the disease management programs using an adjusted version of the relational coordination instrument at T2. This instrument was originally developed for the airline industry [34] and has also been used in hospital [35, 36], primary care [37, 38], and community care [39] settings. These studies investigated relational coordination among professionals (i.e., the quality of communication and relationships among health care professionals) but these studies did not include patients. In the current study, this instrument was used to measure patients’ perceptions of their interactions with health care professionals (i.e., relational coproduction [17]) involved in the disease management programs. This instrument contains three items assessing the quality (frequency, accuracy, and problem-solving nature) of communication with health care professionals and two items concerning relationship dimensions (shared goals and mutual respect) (see Appendix 1 for instrument items). Responses are structured by a four-point scale. Cronbach’s alpha of the instrument was 0.95, indicating excellent reliability.

The short (11-item) version of the Patient Assessment of Chronic Illness Care (PACIC-S) was used to assess patients’ perceptions of the quality of chronic care delivery at T2 [40]. Although the PACIC-S was originally validated with a five-point scale, a four-point scale (ranging from 1 to 4, with higher scores indicating better quality of care) was used to assess the quality of chronic care in 2012 in this study. Cronbach’s alpha of the PACIC-S was 0.88, indicating good reliability.

Research showed that improving care processes and outcomes for chronically ill patients requires implementation of multicomponent interventions, such as disease management programs based on the chronic care model [41]. The chronic care model, however, incorporates flexibility in the implementation of interventions in disease management programs. Thus, such programs may incorporate the elements of the chronic care model to various extents using diverse constellations of interventions. Therefore, a template based on the chronic care model was developed for the collection of data on various interventions implemented within the disease management programs to improve care for chronically ill patients. Project leaders were asked about the implementation of interventions and their experiences with improving patient outcomes. In appendix 2 a full overview is given of all interventions implemented within the 18 disease management programs. Earlier research showed that a disease management program is considered to be based on the CCM if they implement interventions that can be mapped to at least four elements of the CCM [5, 41]. The total number of interventions implemented within each disease management program in the current study ranged from 13 to 43 [42]. All disease management programs implemented interventions in at least four out of the six CCM dimensions and can therefore be considered to be based on the CCM [5, 41, 42]. We scored those programs using at least 34 (which is 60 % of a total of 56 potential interventions) disease management interventions and implementing interventions within all six CCM dimensions as *high-quality* of care (1) versus those programs that implemented fewer disease management interventions (0). Based on this criteria, 33 % of the DMPs are considered to be ‘high-quality’ disease management programs [42]. We used this objective indicator of care quality which is based on the number of interventions implemented within each disease management program.

We also asked participants to provide information on background characteristics, such as age, gender, marital status, and educational level. Patients’ educational levels were characterized using six levels ranging from 1 [no school or primary education (≤7 years)] to 6 [university degree (≥18 years)]. We dichotomized this item into low (no school or primary education) and high (more than primary education) educational levels.

### Statistical analyses

Descriptive statistics were used to describe the study population, patients’ self-management abilities, and their assessments of the quality of chronic care and interactions with health care professionals. Then, we employed correlation analyses to investigate associations among individual characteristics, quality of chronic care, productive patient–professional interaction, and chronically ill patients’ self-management abilities to maintain overall well-being (using Spearman rank or Pearson r when appropriate). Finally, we used a multilevel random-effects model (with patients as level 1 nested in the disease management programs as level 2) to investigate the predictive roles of patients’ background characteristics (step 1), the aggregated variable quality of chronic care delivery (step 2), and productive patient–professional interaction (step 3) in patients’ self-management abilities to maintain overall well-being while controlling for patients’ self-management abilities 1 year previously. All independent variables were standardized. Results were considered statistically significant when two-sided *p* values were ≤0.05.

## Results

Table 1 displays the characteristics of the 1279 patients who completed questionnaires at both T1 and T2. About half (45 %) of the respondents were female, 38 % had a low educational level, and 31 % were single. Respondents’ mean age was 67.62 ± 10.03 (16–94) years. We found that self-management abilities to maintain well-being deteriorated significantly over time (4.18 ± 0.80 at T1 *vs.* 3.98 ± 0.74 at T2; *p* < 0.001).

**Table 1:** Descriptive statistics of patients participating in disease management programs in the Netherlands

	Mean ± standard deviation (range) *or* percentage
Age (years)	67.62 ± 10.03 (16–94)
Gender (female)	45 %
Marital status (single)	31 %
Low educational level	38 %
Patients’ perceptions of chronic care quality (T2)	2.13 ± 0.71 (1–4)
High-quality care (based on implemented interventions)	33 %
Productive patient–professional interaction (T2)	2.93 ± 0.71 (1–4)
Self-management abilities (T1)	4.18 ± 0.80 (1–6)
Self-management abilities (T2)	3.98 ± 0.74 (1–6)

Analyses included only respondents who filled in questionnaires at both T1 and T2 (*n* = 1279)

Associations among individual characteristics, quality of chronic care, productive patient–professional interaction, and self-management abilities are displayed in Table 2. The self-management abilities of patients at T2 were significantly related to self-management abilities at T1 (*r* = 0.74; at *p* < 0.001), age (*r* = −0.10 at *p* = 0.001), single status (*r* = −0.10 at *p* < 0.001), low educational level (*r* = −0.15 at *p* < 0.001), patients’ perceptions of chronic care quality (*r* = 0.16 at *p* < 0.001), high quality of care (based on implemented interventions; *r* = 0.10 at *p* < 0.001), and productivity of interaction with health care professionals (*r* = 0.22 at *p* < 0.001).

**Table 2:** Associations with self-management abilities at T2

	Self-management abilities at T2
Self-management abilities (T1)	0.74^***^
Age	−0.10^***^
Marital status (single)	−0.10^***^
Low educational level	−0.15^***^
Gender (female)	0.04
Patients’ perceptions of chronic care quality (T2)	0.16^***^
High-quality care (based on implemented interventions)	0.10^***^
Productive patient–professional interaction (T2)	0.22^***^

Analyses included only respondents who filled in questionnaires at both T1 and T2 (*n* = 1279)

^***^ *p* ≤0.001 (two-tailed)

Table 3 displays the results of the multilevel analyses. After controlling for self-management abilities at T1, a negative relationship was found between older age (*p* < 0.01), single status (*p* < 0.05), and low educational level (*p* < 0.001). When quality of care delivery was included in the equation (step 2), perceived (*p* < 0.01) and objective (*p* < 0.05) measures of this variable were found to predict patients’ self-management abilities to maintain overall well-being. The quality of care delivery did not mediate the relationships between background characteristics and patients’ self-management abilities. In step 3 of the model, we added productive patient–professional interaction to the equation and found that it predicted chronically ill patients’ self-management abilities (*p* < 0.001) and mediated the relationship between these abilities and the perceived quality of chronic care. The relationship between patients’ perceptions of chronic care quality and their self-management abilities was no longer significant. Patient–professional interaction did not mediate the relationships between background characteristics and self-management abilities. We also found that objective quality of care delivery, age, marital status, and educational level predicted self-management abilities at T2 (in analyses controlling for self-management abilities at T1).

**Table 3:** Predictors of self-management abilities at T2, as assessed by stepwise multilevel regression analyses (random intercepts model, *n* = 1041)

	*Step 1*		*Step 2*		*Step 3*	
	*β*	SE	*β*	SE	*β*	SE
*Step 1*						
Constant	3.98^***^	0.02	3.98^***^	0.02	3.98^***^	0.02
Self-management abilities (T1)	0.54^***^	0.02	0.54^***^	0.02	0.54^***^	0.02
Age	−0.04^**^	0.02	−0.03^*^	0.02	−0.04^*^	0.02
Marital status (single)	−0.04^*^	0.02	−0.04^*^	0.02	−0.03^*^	0.02
Low educational level	−0.06^***^	0.02	−0.07^***^	0.02	−0.07^***^	0.02
Gender (female)	−0.01	0.02	−0.01	0.02	−0.01	0.02
*Step 2*						
Patients’ perceptions of chronic care quality (T2)			0.05^**^	0.02	0.01	0.02
High-quality care (based on implemented interventions)			0.04^*^	0.02	0.04^*^	0.02
*Step 3*						
Productive patient–professional interaction (T2)					0.09^***^	0.02

Multilevel analyses included only respondents who filled in questionnaires at both T1 and T2 (*n* = 1279). Listwise deletion of missing cases resulted in the inclusion of 1041 cases

*SE* Standard error

^***^ *p* <0.001

^**^ *p* <0.01

^*^ *p* <0.05 (two-tailed)

## Discussion

This study aimed to identify relationships among background characteristics, quality of care, productive patient–professional interaction, and self-management abilities to maintain well-being among chronically ill patients. It showed that patients’ experiences with the quality of care, objectively measured care quality, and productive patient–professional interaction were related to chronically ill patients’ self-management abilities. Furthermore, productive interaction was found to mediate the relationship between perceived quality of chronic care delivery and patients’ self-management abilities. These findings underscore the importance of investing in high-quality interaction, characterized by high-quality communication between patients and professionals and the establishment of relationships based on shared goals, knowledge, and mutual respect while taking patients’ preferences into account. Objectively measured quality of care was also a consistently significant predictor of patients’ self-management abilities. Thus, improvement of the quality of chronic care delivery and productivity of patient–professional interaction is expected to protect chronically ill patients’ self-management abilities to maintain overall well-being. The effects of such improvements are limited, however, as most disease management occurs outside of health care practices, beyond professionals’ awareness or involvement. Similar to earlier studies [43–45] it was therefore not surprising that chronically ill patients’ self-management abilities reduced over time. This calls for the need to better understand the components of care delivery that do support self-management skills within the primary care setting. Chronically ill patients control more than 95 % of time spent on disease self-management on a regular, day-to-day basis, making moment-to-moment decisions that accumulate to form patterns in their self-management abilities to maintain well-being [4]. These abilities are thus expected to depend on individual situations and social contexts beyond the influence of health care professionals. Patient’s social network and their involvement in the work of supporting management of long-term conditions have indeed been identified to be important [46, 47]. It may therefore be beneficial to focus on harnessing and sustaining the capacity of social networks to better support their long term illness management [46].

This study found that self-management abilities of patients were negatively related to and significantly predicted by low educational level, single status, and older age, despite the mediating role of productive patient–professional interaction in the association between these abilities and patients’ perceptions of care quality. These findings suggest that patient–professional interaction is not yet sufficiently productive to successfully protect against the deterioration of self-management abilities in some patient groups. As high-quality care delivery is known to be more difficult to achieve among older and less-educated patients, perhaps due to differences in professionals’ behavior toward different patient groups or the demands of these groups, care provision should also take patients’ background characteristics into account [27, 28]. Our findings support this notion and show that it may also apply to patient–professional interaction. Care delivery and interaction should be tailored to each patient’s needs and abilities, to ensure that patients are sufficiently knowledgeable to construct informed preferences and professionals have sufficient knowledge of patients beyond their diseases, including their habits, culture, work, family, and social life [20]. This type of relationship may be more difficult to establish with less-educated, older, and single patients. The core of patient-centered care is the understanding of whether decisions or options correspond to the patient’s values [22]. Some patients, for example, benefit more from individual treatment, whereas group sessions are more effective for others; very motivated and knowledgeable patients need only to be enrolled in a certain treatment program, whereas others require more information and motivation. These differences should be taken into account.

An important limitation of this study is the lack of a control group, which prevented us from determining whether the observed reduction in self-management abilities to maintain well-being over time was smaller than that occurring among chronically ill patients not enrolled in disease management programs based on the CCM. We did find that patients smoked less and were more physically active [48]. Another limitation of the study is the relatively low-response rate especially at the combined time-points. We only found some difference however between respondents who completed questionnaires only at T1 and those who also completed follow-up questionnaires (T1 and T2). Respondents who completed questionnaires at both time points were on average more often single than those who provided responses only at T1, pointing to non-response bias in this respect. While being single was not related to self-management abilities at T1 and objective quality of care we did find significant associations with productive patient-professional interaction (*r* = − 0.07 at *p* <0.05) and self-management abilities at T2 (*r* = − 0.10 at *p* <0.001). No difference was found in self-management abilities, assessment of quality of care, coproduction of care, age, gender or educational level of respondents. The strengths of this study include the investigation of patients’ background characteristics, the quality of care, productivity of interaction, and their effects on the self-management of well-being in diverse patient populations, including those with cardiovascular conditions, COPD, diabetes, heart failure, and comorbidity.

## Conclusion

We can conclude that high-quality care delivery and productive patient–professional interaction seem to be important to protect against further deterioration of chronically ill patients’ self-management abilities to maintain well-being, and that productive interaction may mediate the relationship between perceived quality of care and self-management abilities. According to relational coordination theory patient–professional interaction refers to specific dimensions of relationships and communication aspects that are integral to productive interactions. Within this framework, productive patient–professional interaction occurs through frequent, high-quality communication that is supported by relationships based on shared goals, shared knowledge, and mutual respect enables organizations to better achieve their desired outcomes [16, 17, 37]. Improvement of the quality of chronic care delivery should thus always be accompanied by investment in high-quality communication and relationships between patients and professionals. To foster productive interaction, communication should be accurate, frequent, and focused on problem solving. Patient–professional relationships should be respectful and based on shared goals that account for patients’ preferences and values. These improvements are important first steps in the quest to provide more patient-centered care and stimulate more favorable patient outcomes by improving patients’ self-management abilities. Implementation and actual investments made by professionals in these areas may be ensured through regular measurement and feedback on these issues and using an internal benchmark measuring levels of communication and relationships overtime for further improvements.

## Additional files

Additional file 1:
**Response rate in each disease management program.** (DOCX 14 kb) Additional file 2:
**Instrument to assess relational coproduction of care.** (DOCX 11 kb) Additional file 3:
**Interventions within each disease management program (source Cramm JM, Nieboer AP.** A longitudinal study to identify the influence of quality of chronic care delivery on productive interactions between patients and (teams of) healthcare professionals within disease management programmes. BMJOpen 2014;4:e005914. doi:10.1136/bmjopen-2014-005914). (DOCX 102 kb)
