# Supplementary material for: Chronically ill patients’ self-management abilities to maintain overall well-being: what is needed to take the next step in the primary care setting?
Source: BMC Fam Pract. 2015 Sep 15;16:123. doi: 10.1186/s12875-015-0340-8 (PMC4571068; doi:10.1186/s12875-015-0340-8)
Supplement: Additional file 1: — Response rate in each disease management program. (DOCX 14 kb) [file 12875_2015_340_MOESM1_ESM.docx]

### Additional file 1 Response rate in each disease management program

|  |  | T1 2011 | | T2 2012 | |
| --- | --- | --- | --- | --- | --- |
|  |  | Sent  n | response  % | Sent  n | response  % |
| Onze Lieve Vrouwe Gasthuis (CV-DMP) |  | 369 | 33% | 322 | 25% |
| De Stichting Eerstelijns Samenwerkingsverband Achterveld  (CV-DMP) |  | 134 | 65% | 107 | 65% |
| Regionale Organisatie Huisartsen Amsterdam  (CV-DMP) |  | 275 | 32% | 175 | 28% |
| De Stichting Gezondheidscentra Eindhoven  (CV-DMP) |  | 285 | 42% | 269 | 36% |
| Gezondheidscentrum Maarssenbroek  (CV-DMP) |  | 426 | 48% | 392 | 30% |
| Rijnstate (CV-DMP) |  | 360 | 68% | 338 | 58% |
| Medisch Centrum Oud‐West (CV-DMP) |  | 71 | 35% | 71 | 42% |
| Universiteit Medisch Centrum St. Radboud  (CV-DMP) |  | 250 | 33% | 180 | 27% |
| Wijkgezondheidscentra Huizen (CV-DMP) |  | 440 | 42% | 397 | 39% |
| HAFANK (Hartfalen Noord Kennemerland)  (Heart failure DMP) |  | 49 | 73% | 48 | 35% |
| Huisartsencoöperatie Midden‐Brabant  (COPD DMP) |  | 389 | 54% | 368 | 51% |
| Archiatros (COPD DMP) |  | 522 | 67% | 503 | 45% |
| Stichting Gezond Monnickendam  (COPD DMP) |  | 125 | 62% | 117 | 55% |
| Zorggroep Almere (COPD DMP) |  | 69 | 67% | 67 | 83% |
| Huisartsen Coöperatie Zeist  (Diabetes DMP) |  | 221 | 52% | 204 | 56% |
| Zorggroep Haaglanden (Diabetes DMP) |  | 119 | 46% | 50 | 42% |
| Gezondheidscentrum De Roerdomp  (Diabetes DMP) |  | 259 | 34% | 220 | 41% |
| Chronische Ketenzorg Land van Cuijk en Noord Limburg BV (Co morbidity DMP) |  | 339 | 45% | 306 | 48% |
| **Total of all DMPs** |  | **4702** | **47%** | **4134** | **42%** |

Notes: DMP, Disease Management Programme.
