# Supplementary material for: Chronically ill patients’ self-management abilities to maintain overall well-being: what is needed to take the next step in the primary care setting?
Source: BMC Fam Pract. 2015 Sep 15;16:123. doi: 10.1186/s12875-015-0340-8 (PMC4571068; doi:10.1186/s12875-015-0340-8)
Supplement: Additional file 2: — Instrument to assess relational coproduction of care. (DOCX 11 kb) [file 12875_2015_340_MOESM2_ESM.docx]

**Additional file 2: Instrument to assess relational coproduction of care**

| 1. How **frequently** do you communicate with the following professionals:  - General practitioner never / sometimes / often / always  - Practice nurse never / sometimes / often / always  - Dietician never / sometimes / often / always  - Physical therapist never / sometimes / often / always  - Medical specialist never / sometimes / often / always  - Nurse never / sometimes / often / always |
| --- |
| 2. Do the following professionals communicate **accurately** with you?  - General practitioner never / sometimes / often / always  - Practice nurse never / sometimes / often / always  - Dietician never / sometimes / often / always  - Physical therapist never / sometimes / often / always  - Medical specialist never / sometimes / often / always  - Nurse never / sometimes / often / always |
| 3. When there is a problem do these professionals work together with you to **solve the problem**?  - General practitioner never / sometimes / often / always  - Practice nurse never / sometimes / often / always  - Dietician never / sometimes / often / always  - Physical therapist never / sometimes / often / always  - Medical specialist never / sometimes / often / always  - Nurse never / sometimes / often / always |
| 4. Do these professionals **respect** you?  - General practitioner never / sometimes / often / always  - Practice nurse never / sometimes / often / always  - Dietician never / sometimes / often / always  - Physical therapist never / sometimes / often / always  - Medical specialist never / sometimes / often / always  - Nurse never / sometimes / often / always |
| 5. Do these professionals **share your goals**? |

- General practitioner never / sometimes / often / always

- Practice nurse never / sometimes / often / always

- Dietician never / sometimes / often / always

- Physical therapist never / sometimes / often / always

- Medical specialist never / sometimes / often / always

- Nurse never / sometimes / often / always
