# Supplementary material for: Chronically ill patients’ self-management abilities to maintain overall well-being: what is needed to take the next step in the primary care setting?
Source: BMC Fam Pract. 2015 Sep 15;16:123. doi: 10.1186/s12875-015-0340-8 (PMC4571068; doi:10.1186/s12875-015-0340-8)
Supplement: Additional file 3: — Interventions within each disease management program (source Cramm JM, Nieboer AP. A longitudinal study to identify the influence of quality of chronic care delivery on productive interactions between patients and (teams of) healthcare professionals within disease management programmes. BMJOpen 2014;4:e005914. doi:10.1136/bmjopen-2014-005914). (DOCX 102 kb) [file 12875_2015_340_MOESM3_ESM.docx]

**Additional file 3: Interventions within each disease management program**

*(source Cramm JM, Nieboer AP. A longitudinal study to identify the influence of quality of chronic care*

*delivery on productive interactions between patients and (teams of) healthcare professionals within disease management programmes. BMJOpen 2014;4:e005914. doi:10.1136/bmjopen-2014-005914)*

| **Cardiovascular disease management program: Onze Lieve Vrouwe Gasthuis / low quality of care***  * Based on the criteria of scoring programs using at least 34 interventions and implementing interventions within all six CCM dimensions as high-quality of care (1) versus those programs that implemented fewer disease management interventions (0) | | **Existing**  **/implemented interventions** |
| --- | --- | --- |
| Organizational support | Integrated financing | - |
| Organizational support | Specific policies and subsidies for foreign population | - |
| Organizational support | Sustainable financing agreements with health insurers | - |
| Community | Communication platform | - |
| Community | Health market | - |
| Community | Cooperation with external community partners | - |
| Community | Multidisciplinary and transmural collaboration | - |
| Community | Role model in the area | **√** |
| Community | Regional collaboration for spread of the DMP | **√** |
| Community | Treatment and care pathways in out- and inpatient care | **√** |
| Community | Involvement of patient groups and/or panels in care design | - |
| Community | Regional training course | **√** |
| Community | Family participation | - |
| Self management | Promotion of disease specific information | **√** |
| Self management | Individual care plan | **√** |
| Self management | Life-style interventions (physical activity, diet, quit smoking) | - |
| Self management | Support of self-management (e.g. email or sms, e-consult) | - |
| Self management | Tele-monitoring | - |
| Self management | Personal coaching | **√** |
| Self management | Motivational interviewing | **√** |
| Self management | Informational meetings | - |
| Self management | Diagnosis and treatment of mental health issues | **√** |
| Self management | Reflection meetings | - |
| Self management | Group sessions for patients and family | - |
| Self management | Cognitive behavioural therapy | **-** |
| Decision Support | Care standards / Clinical guidelines | **√** |
| Decision Support | Uniform treatment protocol in outpatient and inpatient care | **√** |
| Decision Support | Training and independence of practise assistants | **√** |
| Decision Support | Professional education and training for care providers | **√** |
| Decision Support | Automatic measurement of process/outcome indicators | **-** |
| Decision Support | Care protocols for immigrants | - |
| Decision Support | Audit and feedback | **√** |
| Decision Support | Periodic evaluation of interventions and goal achievement | - |
| Decision Support | Structural participation in knowledge exchange | - |
| Decision Support | Quality of Life questionnaire | - |
| Decision Support | Qualitative evaluation of care via focus-groups with patients | - |
| Decision Support | Measurement of patient satisfaction | **√** |
| Delivery System Design | Delegation of care from specialist to nurse/care practitioner | **√** |
| Delivery System Design | Substitution of inpatient with outpatient care | **√** |
| Delivery System Design | Systematic follow-up of patients | **√** |
| Delivery System Design | One-stop outpatient clinic | - |
| Delivery System Design | Specific plan for immigrant population | - |
| Delivery System Design | Meetings of different disciplines for exchanging information | - |
| Delivery System Design | Monitoring of high-risk patients | **√** |
| Delivery System Design | Board of clients | **√** |
| Delivery System Design | Periodic discussions between professionals (and patients) | **√** |
| Delivery System Design | Expansion of chain care to the secondary care setting | - |
| Delivery System Design | Joint consultation hours | - |
| Delivery System Design | Stepped care method | - |
| ICT | Electronic Patient Records system with Patient Portal | - |
| ICT | Hospital or Practice Information System | **√** |
| ICT | Integrated Chain Information System | - |
| ICT | Use of ICT for Internal and/or regional benchmarking | - |
| ICT | Create a safe environment for data exchange | - |
| ICT | Systematic registration by every caregiver | - |
| ICT | Exchange of information between different care disciplines | - |
| **Cardiovascular disease management program: De Stichting Eerstelijns Samenwerkingsverband Achterveld / low quality of care***  * Based on the criteria of scoring programs using at least 34 interventions and implementing interventions within all six CCM dimensions as high-quality of care (1) versus those programs that implemented fewer disease management interventions (0) | | **Existing**  **/implemented interventions** |
| Organizational support | Integrated financing | - |
| Organizational support | Specific policies and subsidies for foreign population | - |
| Organizational support | Sustainable financing agreements with health insurers | - |
| Community | Communication platform | - |
| Community | Health market | - |
| Community | Cooperation with external community partners | **√** |
| Community | Multidisciplinary and transmural collaboration | **√** |
| Community | Role model in the area | - |
| Community | Regional collaboration for spread of the DMP | - |
| Community | Treatment and care pathways in out- and inpatient care | **√** |
| Community | Involvement of patient groups and/or panels in care design | - |
| Community | Regional training course | - |
| Community | Family participation | - |
| Self management | Promotion of disease specific information | **√** |
| Self management | Individual care plan | **√** |
| Self management | Life-style interventions (physical activity, diet, quit smoking) | **√** |
| Self management | Support of self-management (e.g. email or sms, e-consult) | - |
| Self management | Tele-monitoring | - |
| Self management | Personal coaching | **√** |
| Self management | Motivational interviewing | **√** |
| Self management | Informational meetings | - |
| Self management | Diagnosis and treatment of mental health issues | - |
| Self management | Reflection meetings | - |
| Self management | Group sessions for patients and family | - |
| Self management | Cognitive behavioural therapy | - |
| Decision Support | Care standards / Clinical guidelines | **√** |
| Decision Support | Uniform treatment protocol in outpatient and inpatient care | - |
| Decision Support | Training and independence of practise assistants | **√** |
| Decision Support | Professional education and training for care providers | **√** |
| Decision Support | Automatic measurement of process/outcome indicators | **√** |
| Decision Support | Care protocols for immigrants | - |
| Decision Support | Audit and feedback | - |
| Decision Support | Periodic evaluation of interventions and goal achievement | - |
| Decision Support | Structural participation in knowledge exchange | - |
| Decision Support | Quality of Life questionnaire | **√** |
| Decision Support | Qualitative evaluation of care via focus-groups with patients | - |
| Decision Support | Measurement of patient satisfaction | **√** |
| Delivery System Design | Delegation of care from specialist to nurse/care practitioner | **√** |
| Delivery System Design | Substitution of inpatient with outpatient care | **√** |
| Delivery System Design | Systematic follow-up of patients | **√** |
| Delivery System Design | One-stop outpatient clinic | - |
| Delivery System Design | Specific plan for immigrant population | - |
| Delivery System Design | Expansion of chain care to the secondary care setting | - |
| Delivery System Design | Joint consultation hours | - |
| Delivery System Design | Meetings of different disciplines for exchanging information | **√** |
| Delivery System Design | Monitoring of high-risk patients | **√** |
| Delivery System Design | Board of clients | - |
| Delivery System Design | Periodic discussions between professionals (and patients) | - |
| Delivery System Design | Stepped care method | - |
| ICT | Electronic Patient Records system with Patient Portal | - |
| ICT | Hospital or Practice Information System | **√** |
| ICT | Integrated Chain Information System | **√** |
| ICT | Use of ICT for Internal and/or regional benchmarking | - |
| ICT | Create a safe environment for data exchange | **√** |
| ICT | Systematic registration by every caregiver | **√** |
| ICT | Exchange of information between different care disciplines | **√** |

| **Cardiovascular disease management program: Regionale Organisatie Huisartsen Amsterdam / low quality of care***  * Based on the criteria of scoring programs using at least 34 interventions and implementing interventions within all six CCM dimensions as high-quality of care (1) versus those programs that implemented fewer disease management interventions (0) | | **Existing**  **/implemented interventions** |
| --- | --- | --- |
| Organizational support | Integrated financing | - |
| Organizational support | Specific policies and subsidies for foreign population | **√** |
| Organizational support | Sustainable financing agreements with health insurers | - |
| Community | Communication platform | - |
| Community | Health market | - |
| Community | Cooperation with external community partners | **√** |
| Community | Multidisciplinary and transmural collaboration | **√** |
| Community | Role model in the area | **√** |
| Community | Regional collaboration for spread of the DMP | **√** |
| Community | Treatment and care pathways in out- and inpatient care | **√** |
| Community | Involvement of patient groups and/or panels in care design | **√** |
| Community | Regional training course | **√** |
| Community | Family participation | - |
| Self management | Promotion of disease specific information | **√** |
| Self management | Individual care plan | - |
| Self management | Life-style interventions (physical activity, diet, quit smoking) | **√** |
| Self management | Support of self-management (e.g. email or sms, e-consult) | - |
| Self management | Tele-monitoring | - |
| Self management | Personal coaching | **√** |
| Self management | Motivational interviewing | - |
| Self management | Informational meetings | - |
| Self management | Diagnosis and treatment of mental health issues | - |
| Self management | Reflection meetings | - |
| Self management | Group sessions for patients and family | - |
| Self management | Cognitive behavioural therapy | - |
| Decision Support | Care standards / Clinical guidelines | **√** |
| Decision Support | Uniform treatment protocol in outpatient and inpatient care | **√** |
| Decision Support | Training and independence of practise assistants | **√** |
| Decision Support | Professional education and training for care providers | **√** |
| Decision Support | Automatic measurement of process/outcome indicators | **√** |
| Decision Support | Care protocols for immigrants | - |
| Decision Support | Audit and feedback | **√** |
| Decision Support | Periodic evaluation of interventions and goal achievement | - |
| Decision Support | Structural participation in knowledge exchange | - |
| Decision Support | Quality of Life questionnaire | - |
| Decision Support | Qualitative evaluation of care via focus-groups with patients | - |
| Decision Support | Measurement of patient satisfaction | - |
| Delivery System Design | Delegation of care from specialist to nurse/care practitioner | **√** |
| Delivery System Design | Substitution of inpatient with outpatient care | - |
| Delivery System Design | Systematic follow-up of patients | **√** |
| Delivery System Design | One-stop outpatient clinic | - |
| Delivery System Design | Specific plan for immigrant population | **√** |
| Delivery System Design | Expansion of chain care to the secondary care setting | **√** |
| Delivery System Design | Joint consultation hours | - |
| Delivery System Design | Meetings of different disciplines for exchanging information | **√** |
| Delivery System Design | Monitoring of high-risk patients | - |
| Delivery System Design | Board of clients | - |
| Delivery System Design | Periodic discussions between professionals (and patients) | **√** |
| Delivery System Design | Stepped care method | **√** |
| ICT | Electronic Patient Records system with Patient Portal | - |
| ICT | Hospital or Practice Information System | **√** |
| ICT | Integrated Chain Information System | **√** |
| ICT | Use of ICT for Internal and/or regional benchmarking | **√** |
| ICT | Create a safe environment for data exchange | - |
| ICT | Systematic registration by every caregiver | **√** |
| ICT | Exchange of information between different care disciplines | **√** |

| **Cardiovascular disease management program: De Stichting Gezondheidscentra Eindhoven / high quality of care***  * Based on the criteria of scoring programs using at least 34 interventions and implementing interventions within all six CCM dimensions as high-quality of care (1) versus those programs that implemented fewer disease management interventions (0) | | **Existing**  **/implemented interventions** |
| --- | --- | --- |
| Organizational support | Integrated financing | **√** |
| Organizational support | Specific policies and subsidies for foreign population | **√** |
| Organizational support | Sustainable financing agreements with health insurers | **√** |
| Community | Communication platform | - |
| Community | Health market | - |
| Community | Cooperation with external community partners | **√** |
| Community | Multidisciplinary and transmural collaboration | **√** |
| Community | Role model in the area | - |
| Community | Regional collaboration for spread of the DMP | **√** |
| Community | Treatment and care pathways in out- and inpatient care | **√** |
| Community | Involvement of patient groups and/or panels in care design | **√** |
| Community | Regional training course | **√** |
| Community | Family participation | **√** |
| Self management | Promotion of disease specific information | **√** |
| Self management | Individual care plan | **√** |
| Self management | Life-style interventions (physical activity, diet, quit smoking) | **√** |
| Self management | Support of self-management (e.g. email or sms, e-consult) | - |
| Self management | Tele-monitoring | - |
| Self management | Personal coaching | **√** |
| Self management | Motivational interviewing | **√** |
| Self management | Informational meetings | - |
| Self management | Diagnosis and treatment of mental health issues | **√** |
| Self management | Reflection meetings | - |
| Self management | Group sessions for patients and family | - |
| Self management | Cognitive behavioural therapy | - |
| Decision Support | Care standards / Clinical guidelines | **√** |
| Decision Support | Uniform treatment protocol in outpatient and inpatient care | **√** |
| Decision Support | Training and independence of practise assistants | **√** |
| Decision Support | Professional education and training for care providers | **√** |
| Decision Support | Automatic measurement of process/outcome indicators | **√** |
| Decision Support | Care protocols for immigrants | **√** |
| Decision Support | Audit and feedback | **√** |
| Decision Support | Periodic evaluation of interventions and goal achievement | **√** |
| Decision Support | Structural participation in knowledge exchange | **√** |
| Decision Support | Quality of Life questionnaire | **√** |
| Decision Support | Qualitative evaluation of care via focus-groups with patients | **√** |
| Decision Support | Measurement of patient satisfaction | **√** |
| Delivery System Design | Delegation of care from specialist to nurse/care practitioner | **√** |
| Delivery System Design | Substitution of inpatient with outpatient care | - |
| Delivery System Design | Systematic follow-up of patients | **√** |
| Delivery System Design | One-stop outpatient clinic | **√** |
| Delivery System Design | Specific plan for immigrant population | **√** |
| Delivery System Design | Expansion of chain care to the secondary care setting | - |
| Delivery System Design | Joint consultation hours | - |
| Delivery System Design | Meetings of different disciplines for exchanging information | **√** |
| Delivery System Design | Monitoring of high-risk patients | **√** |
| Delivery System Design | Board of clients | **√** |
| Delivery System Design | Periodic discussions between professionals (and patients) | **√** |
| Delivery System Design | Stepped care method | **√** |
| ICT | Electronic Patient Records system with Patient Portal | - |
| ICT | Hospital or Practice Information System | **√** |
| ICT | Integrated Chain Information System | **√** |
| ICT | Use of ICT for Internal and/or regional benchmarking | **√** |
| ICT | Create a safe environment for data exchange | **√** |
| ICT | Systematic registration by every caregiver | **√** |
| ICT | Exchange of information between different care disciplines | **√** |

| **Cardiovascular disease management program: Gezondheidscentrum Maarssenbroek / low quality of care***  * Based on the criteria of scoring programs using at least 34 interventions and implementing interventions within all six CCM dimensions as high-quality of care (1) versus those programs that implemented fewer disease management interventions (0) | | **Existing**  **/implemented interventions** |
| --- | --- | --- |
| Organizational support | Integrated financing | - |
| Organizational support | Specific policies and subsidies for foreign population | - |
| Organizational support | Sustainable financing agreements with health insurers | **√** |
| Community | Communication platform | - |
| Community | Health market | - |
| Community | Cooperation with external community partners | **√** |
| Community | Multidisciplinary and transmural collaboration | **√** |
| Community | Role model in the area | - |
| Community | Regional collaboration for spread of the DMP | - |
| Community | Treatment and care pathways in out- and inpatient care | **√** |
| Community | Involvement of patient groups and/or panels in care design | - |
| Community | Regional training course | **√** |
| Community | Family participation | - |
| Self management | Promotion of disease specific information | - |
| Self management | Individual care plan | **√** |
| Self management | Life-style interventions (physical activity, diet, quit smoking) | **√** |
| Self management | Support of self-management (e.g. email or sms, e-consult) | **√** |
| Self management | Tele-monitoring | - |
| Self management | Personal coaching | **√** |
| Self management | Motivational interviewing | **√** |
| Self management | Informational meetings | **√** |
| Self management | Diagnosis and treatment of mental health issues | - |
| Self management | Reflection meetings | - |
| Self management | Group sessions for patients and family | - |
| Self management | Cognitive behavioural therapy | - |
| Decision Support | Care standards / Clinical guidelines | **√** |
| Decision Support | Uniform treatment protocol in outpatient and inpatient care | **√** |
| Decision Support | Training and independence of practise assistants | **√** |
| Decision Support | Professional education and training for care providers | **√** |
| Decision Support | Automatic measurement of process/outcome indicators | **√** |
| Decision Support | Care protocols for immigrants | - |
| Decision Support | Audit and feedback | **√** |
| Decision Support | Periodic evaluation of interventions and goal achievement | - |
| Decision Support | Structural participation in knowledge exchange | - |
| Decision Support | Quality of Life questionnaire | - |
| Decision Support | Qualitative evaluation of care via focus-groups with patients | - |
| Decision Support | Measurement of patient satisfaction | **√** |
| Delivery System Design | Delegation of care from specialist to nurse/care practitioner | **√** |
| Delivery System Design | Substitution of inpatient with outpatient care | - |
| Delivery System Design | Systematic follow-up of patients | **√** |
| Delivery System Design | One-stop outpatient clinic | - |
| Delivery System Design | Specific plan for immigrant population | - |
| Delivery System Design | Expansion of chain care to the secondary care setting | - |
| Delivery System Design | Joint consultation hours | - |
| Delivery System Design | Meetings of different disciplines for exchanging information | **√** |
| Delivery System Design | Monitoring of high-risk patients | **√** |
| Delivery System Design | Board of clients | **√** |
| Delivery System Design | Periodic discussions between professionals (and patients) | - |
| Delivery System Design | Stepped care method | - |
| ICT | Electronic Patient Records system with Patient Portal | **√** |
| ICT | Hospital or Practice Information System | **√** |
| ICT | Integrated Chain Information System | **√** |
| ICT | Use of ICT for Internal and/or regional benchmarking | **√** |
| ICT | Create a safe environment for data exchange | - |
| ICT | Systematic registration by every caregiver | **√** |
| ICT | Exchange of information between different care disciplines | **√** |

| **Cardiovascular disease management program: Rijnstate / high quality of care***  * Based on the criteria of scoring programs using at least 34 interventions and implementing interventions within all six CCM dimensions as high-quality of care (1) versus those programs that implemented fewer disease management interventions (0) | | **Existing**  **/implemented interventions** |
| --- | --- | --- |
| Organizational support | Integrated financing | **√** |
| Organizational support | Specific policies and subsidies for foreign population | - |
| Organizational support | Sustainable financing agreements with health insurers | **√** |
| Community | Communication platform | - |
| Community | Health market | - |
| Community | Cooperation with external community partners | **√** |
| Community | Multidisciplinary and transmural collaboration | **√** |
| Community | Role model in the area | **√** |
| Community | Regional collaboration for spread of the DMP | **√** |
| Community | Treatment and care pathways in out- and inpatient care | **√** |
| Community | Involvement of patient groups and/or panels in care design | **√** |
| Community | Regional training course | **√** |
| Community | Family participation | - |
| Self management | Promotion of disease specific information | **√** |
| Self management | Individual care plan | **√** |
| Self management | Life-style interventions (physical activity, diet, quit smoking) | **√** |
| Self management | Support of self-management (e.g. email or sms, e-consult) | - |
| Self management | Tele-monitoring | - |
| Self management | Personal coaching | **√** |
| Self management | Motivational interviewing | **√** |
| Self management | Informational meetings | - |
| Self management | Diagnosis and treatment of mental health issues | - |
| Self management | Reflection meetings | - |
| Self management | Group sessions for patients and family | **√** |
| Self management | Cognitive behavioural therapy | - |
| Decision Support | Care standards / Clinical guidelines | **√** |
| Decision Support | Uniform treatment protocol in outpatient and inpatient care | **√** |
| Decision Support | Training and independence of practise assistants | **√** |
| Decision Support | Professional education and training for care providers | **√** |
| Decision Support | Automatic measurement of process/outcome indicators | **√** |
| Decision Support | Care protocols for immigrants | - |
| Decision Support | Audit and feedback | - |
| Decision Support | Periodic evaluation of interventions and goal achievement | - |
| Decision Support | Structural participation in knowledge exchange | - |
| Decision Support | Quality of Life questionnaire | - |
| Decision Support | Qualitative evaluation of care via focus-groups with patients | - |
| Decision Support | Measurement of patient satisfaction | **√** |
| Delivery System Design | Delegation of care from specialist to nurse/care practitioner | **√** |
| Delivery System Design | Substitution of inpatient with outpatient care | **√** |
| Delivery System Design | Systematic follow-up of patients | **√** |
| Delivery System Design | One-stop outpatient clinic | **√** |
| Delivery System Design | Specific plan for immigrant population | - |
| Delivery System Design | Expansion of chain care to the secondary care setting | - |
| Delivery System Design | Joint consultation hours | **√** |
| Delivery System Design | Meetings of different disciplines for exchanging information | **√** |
| Delivery System Design | Monitoring of high-risk patients | **√** |
| Delivery System Design | Board of clients | - |
| Delivery System Design | Periodic discussions between professionals (and patients) | **√** |
| Delivery System Design | Stepped care method | - |
| ICT | Electronic Patient Records system with Patient Portal | - |
| ICT | Hospital or Practice Information System | **√** |
| ICT | Integrated Chain Information System | **√** |
| ICT | Use of ICT for Internal and/or regional benchmarking | **√** |
| ICT | Create a safe environment for data exchange | **√** |
| ICT | Systematic registration by every caregiver | **√** |
| ICT | Exchange of information between different care disciplines | **√** |

| **Cardiovascular disease management program: Medisch Centrum Oud-West / low quality of care***  * Based on the criteria of scoring programs using at least 34 interventions and implementing interventions within all six CCM dimensions as high-quality of care (1) versus those programs that implemented fewer disease management interventions (0) | | **Existing**  **/implemented interventions** |
| --- | --- | --- |
| Organizational support | Integrated financing | - |
| Organizational support | Specific policies and subsidies for foreign population | **√** |
| Organizational support | Sustainable financing agreements with health insurers | **√** |
| Community | Communication platform | - |
| Community | Health market | **√** |
| Community | Cooperation with external community partners | - |
| Community | Multidisciplinary and transmural collaboration | - |
| Community | Role model in the area | - |
| Community | Regional collaboration for spread of the DMP | - |
| Community | Treatment and care pathways in out- and inpatient care | - |
| Community | Involvement of patient groups and/or panels in care design | - |
| Community | Regional training course | - |
| Community | Family participation | - |
| Self management | Promotion of disease specific information | **√** |
| Self management | Individual care plan | **√** |
| Self management | Life-style interventions (physical activity, diet, quit smoking) | **√** |
| Self management | Support of self-management (e.g. email or sms, e-consult) | - |
| Self management | Tele-monitoring | - |
| Self management | Personal coaching | **√** |
| Self management | Motivational interviewing | **√** |
| Self management | Informational meetings | - |
| Self management | Diagnosis and treatment of mental health issues | **√** |
| Self management | Reflection meetings | - |
| Self management | Group sessions for patients and family | - |
| Self management | Cognitive behavioural therapy | - |
| Decision Support | Care standards / Clinical guidelines | **√** |
| Decision Support | Uniform treatment protocol in outpatient and inpatient care | - |
| Decision Support | Training and independence of practise assistants | **√** |
| Decision Support | Professional education and training for care providers | **√** |
| Decision Support | Automatic measurement of process/outcome indicators | **√** |
| Decision Support | Care protocols for immigrants | - |
| Decision Support | Audit and feedback | - |
| Decision Support | Periodic evaluation of interventions and goal achievement | **√** |
| Decision Support | Structural participation in knowledge exchange | **√** |
| Decision Support | Quality of Life questionnaire | - |
| Decision Support | Qualitative evaluation of care via focus-groups with patients | - |
| Decision Support | Measurement of patient satisfaction | - |
| Delivery System Design | Delegation of care from specialist to nurse/care practitioner | **√** |
| Delivery System Design | Substitution of inpatient with outpatient care | - |
| Delivery System Design | Systematic follow-up of patients | **√** |
| Delivery System Design | One-stop outpatient clinic | - |
| Delivery System Design | Specific plan for immigrant population | **√** |
| Delivery System Design | Expansion of chain care to the secondary care setting | - |
| Delivery System Design | Joint consultation hours | - |
| Delivery System Design | Meetings of different disciplines for exchanging information | **√** |
| Delivery System Design | Monitoring of high-risk patients | **√** |
| Delivery System Design | Board of clients | - |
| Delivery System Design | Periodic discussions between professionals (and patients) | **√** |
| Delivery System Design | Stepped care method | **√** |
| ICT | Electronic Patient Records system with Patient Portal | - |
| ICT | Hospital or Practice Information System | **√** |
| ICT | Integrated Chain Information System | - |
| ICT | Use of ICT for Internal and/or regional benchmarking | - |
| ICT | Create a safe environment for data exchange | - |
| ICT | Systematic registration by every caregiver | - |
| ICT | Exchange of information between different care disciplines | - |

| **Cardiovascular disease management program: Universiteit Medisch Centrum St. Radboud / low quality of care***  * Based on the criteria of scoring programs using at least 34 interventions and implementing interventions within all six CCM dimensions as high-quality of care (1) versus those programs that implemented fewer disease management interventions (0) | | **Existing**  **/implemented interventions** |
| --- | --- | --- |
| Organizational support | Integrated financing | - |
| Organizational support | Specific policies and subsidies for foreign population | - |
| Organizational support | Sustainable financing agreements with health insurers | - |
| Community | Communication platform | **√** |
| Community | Health market | - |
| Community | Cooperation with external community partners | **√** |
| Community | Multidisciplinary and transmural collaboration | **√** |
| Community | Role model in the area | **√** |
| Community | Regional collaboration for spread of the DMP | - |
| Community | Treatment and care pathways in out- and inpatient care | **√** |
| Community | Involvement of patient groups and/or panels in care design | - |
| Community | Regional training course | - |
| Community | Family participation | - |
| Self management | Promotion of disease specific information | **√** |
| Self management | Individual care plan | **√** |
| Self management | Life-style interventions (physical activity, diet, quit smoking) | **√** |
| Self management | Support of self-management (e.g. email or sms, e-consult) | - |
| Self management | Tele-monitoring | - |
| Self management | Personal coaching | **√** |
| Self management | Motivational interviewing | **√** |
| Self management | Informational meetings | - |
| Self management | Diagnosis and treatment of mental health issues | **√** |
| Self management | Reflection meetings | - |
| Self management | Group sessions for patients and family | - |
| Self management | Cognitive behavioural therapy | - |
| Decision Support | Care standards / Clinical guidelines | **√** |
| Decision Support | Uniform treatment protocol in outpatient and inpatient care | - |
| Decision Support | Training and independence of practise assistants | **√** |
| Decision Support | Professional education and training for care providers | **√** |
| Decision Support | Automatic measurement of process/outcome indicators | - |
| Decision Support | Care protocols for immigrants | - |
| Decision Support | Audit and feedback | **√** |
| Decision Support | Periodic evaluation of interventions and goal achievement | - |
| Decision Support | Structural participation in knowledge exchange | **√** |
| Decision Support | Quality of Life questionnaire | - |
| Decision Support | Qualitative evaluation of care via focus-groups with patients | - |
| Decision Support | Measurement of patient satisfaction | - |
| Delivery System Design | Delegation of care from specialist to nurse/care practitioner | - |
| Delivery System Design | Substitution of inpatient with outpatient care | - |
| Delivery System Design | Systematic follow-up of patients | **√** |
| Delivery System Design | One-stop outpatient clinic | - |
| Delivery System Design | Specific plan for immigrant population | - |
| Delivery System Design | Expansion of chain care to the secondary care setting | **√** |
| Delivery System Design | Joint consultation hours | - |
| Delivery System Design | Meetings of different disciplines for exchanging information | **√** |
| Delivery System Design | Monitoring of high-risk patients | **√** |
| Delivery System Design | Board of clients | - |
| Delivery System Design | Periodic discussions between professionals (and patients) | - |
| Delivery System Design | Stepped care method | - |
| ICT | Electronic Patient Records system with Patient Portal | - |
| ICT | Hospital or Practice Information System | **√** |
| ICT | Integrated Chain Information System | **√** |
| ICT | Use of ICT for Internal and/or regional benchmarking | **√** |
| ICT | Create a safe environment for data exchange | - |
| ICT | Systematic registration by every caregiver | **√** |
| ICT | Exchange of information between different care disciplines | **√** |

| **Cardiovascular disease management program: Wijkgezondheidscentra Huizen / low quality of care***  * Based on the criteria of scoring programs using at least 34 interventions and implementing interventions within all six CCM dimensions as high-quality of care (1) versus those programs that implemented fewer disease management interventions (0) | | **Existing**  **/implemented interventions** |
| --- | --- | --- |
| Organizational support | Integrated financing | - |
| Organizational support | Specific policies and subsidies for foreign population | - |
| Organizational support | Sustainable financing agreements with health insurers | - |
| Community | Communication platform | - |
| Community | Health market | - |
| Community | Cooperation with external community partners | - |
| Community | Multidisciplinary and transmural collaboration | - |
| Community | Role model in the area | - |
| Community | Regional collaboration for spread of the DMP | - |
| Community | Treatment and care pathways in out- and inpatient care | - |
| Community | Involvement of patient groups and/or panels in care design | - |
| Community | Regional training course | - |
| Community | Family participation | - |
| Self management | Promotion of disease specific information | - |
| Self management | Individual care plan | **√** |
| Self management | Life-style interventions (physical activity, diet, quit smoking) | **√** |
| Self management | Support of self-management (e.g. email or sms, e-consult) | - |
| Self management | Tele-monitoring | - |
| Self management | Personal coaching | - |
| Self management | Motivational interviewing | **√** |
| Self management | Informational meetings | - |
| Self management | Diagnosis and treatment of mental health issues | - |
| Self management | Reflection meetings | - |
| Self management | Group sessions for patients and family | - |
| Self management | Cognitive behavioural therapy | - |
| Decision Support | Care standards / Clinical guidelines | **√** |
| Decision Support | Uniform treatment protocol in outpatient and inpatient care | - |
| Decision Support | Training and independence of practise assistants | **√** |
| Decision Support | Professional education and training for care providers | **√** |
| Decision Support | Automatic measurement of process/outcome indicators | - |
| Decision Support | Care protocols for immigrants | - |
| Decision Support | Audit and feedback | - |
| Decision Support | Periodic evaluation of interventions and goal achievement | **√** |
| Decision Support | Structural participation in knowledge exchange | - |
| Decision Support | Quality of Life questionnaire | - |
| Decision Support | Qualitative evaluation of care via focus-groups with patients | - |
| Decision Support | Measurement of patient satisfaction | - |
| Delivery System Design | Delegation of care from specialist to nurse/care practitioner | **√** |
| Delivery System Design | Substitution of inpatient with outpatient care | - |
| Delivery System Design | Systematic follow-up of patients | **√** |
| Delivery System Design | One-stop outpatient clinic | - |
| Delivery System Design | Specific plan for immigrant population | - |
| Delivery System Design | Expansion of chain care to the secondary care setting | - |
| Delivery System Design | Joint consultation hours | - |
| Delivery System Design | Meetings of different disciplines for exchanging information | **√** |
| Delivery System Design | Monitoring of high-risk patients | - |
| Delivery System Design | Board of clients | - |
| Delivery System Design | Periodic discussions between professionals (and patients) | - |
| Delivery System Design | Stepped care method | - |
| ICT | Electronic Patient Records system with Patient Portal | - |
| ICT | Hospital or Practice Information System | **√** |
| ICT | Integrated Chain Information System | - |
| ICT | Use of ICT for Internal and/or regional benchmarking | **√** |
| ICT | Create a safe environment for data exchange | - |
| ICT | Systematic registration by every caregiver | **√** |
| ICT | Exchange of information between different care disciplines | - |

| **Heart failure disease management program: HAFANK (Hartfalen Noord Kennemerland) / low quality of care***  * Based on the criteria of scoring programs using at least 34 interventions and implementing interventions within all six CCM dimensions as high-quality of care (1) versus those programs that implemented fewer disease management interventions (0) | | **Existing**  **/implemented interventions** |
| --- | --- | --- |
| Organizational support | Integrated financing | - |
| Organizational support | Specific policies and subsidies for foreign population | - |
| Organizational support | Sustainable financing agreements with health insurers | - |
| Community | Communication platform | - |
| Community | Health market | - |
| Community | Cooperation with external community partners | **√** |
| Community | Multidisciplinary and transmural collaboration | **√** |
| Community | Role model in the area | - |
| Community | Regional collaboration for spread of the DMP | **√** |
| Community | Treatment and care pathways in out- and inpatient care | **√** |
| Community | Involvement of patient groups and/or panels in care design | - |
| Community | Regional training course | **√** |
| Community | Family participation | **√** |
| Self management | Promotion of disease specific information | **√** |
| Self management | Individual care plan | **√** |
| Self management | Life-style interventions (physical activity, diet, quit smoking) | **√** |
| Self management | Support of self-management (e.g. email or sms, e-consult) | - |
| Self management | Tele-monitoring | - |
| Self management | Personal coaching | **√** |
| Self management | Motivational interviewing | **√** |
| Self management | Informational meetings | **√** |
| Self management | Diagnosis and treatment of mental health issues | - |
| Self management | Reflection meetings | - |
| Self management | Group sessions for patients and family | - |
| Self management | Cognitive behavioural therapy | - |
| Decision Support | Care standards / Clinical guidelines | **√** |
| Decision Support | Uniform treatment protocol in outpatient and inpatient care | - |
| Decision Support | Training and independence of practise assistants | **√** |
| Decision Support | Professional education and training for care providers | **√** |
| Decision Support | Automatic measurement of process/outcome indicators | **√** |
| Decision Support | Care protocols for immigrants | - |
| Decision Support | Audit and feedback | - |
| Decision Support | Periodic evaluation of interventions and goal achievement | - |
| Decision Support | Structural participation in knowledge exchange | - |
| Decision Support | Quality of Life questionnaire | - |
| Decision Support | Qualitative evaluation of care via focus-groups with patients | - |
| Decision Support | Measurement of patient satisfaction | - |
| Delivery System Design | Delegation of care from specialist to nurse/care practitioner | **√** |
| Delivery System Design | Substitution of inpatient with outpatient care | - |
| Delivery System Design | Systematic follow-up of patients | **√** |
| Delivery System Design | One-stop outpatient clinic | - |
| Delivery System Design | Specific plan for immigrant population | - |
| Delivery System Design | Expansion of chain care to the secondary care setting | - |
| Delivery System Design | Joint consultation hours | **√** |
| Delivery System Design | Meetings of different disciplines for exchanging information | **√** |
| Delivery System Design | Monitoring of high-risk patients | **√** |
| Delivery System Design | Board of clients | - |
| Delivery System Design | Periodic discussions between professionals (and patients) | - |
| Delivery System Design | Stepped care method | - |
| ICT | Electronic Patient Records system with Patient Portal | - |
| ICT | Hospital or Practice Information System | **√** |
| ICT | Integrated Chain Information System | **√** |
| ICT | Use of ICT for Internal and/or regional benchmarking | **√** |
| ICT | Create a safe environment for data exchange | - |
| ICT | Systematic registration by every caregiver | **√** |
| ICT | Exchange of information between different care disciplines | **√** |

| **COPD disease management program: Huisartsencoöperatie Midden-Brabant / low quality of care***  * Based on the criteria of scoring programs using at least 34 interventions and implementing interventions within all six CCM dimensions as high-quality of care (1) versus those programs that implemented fewer disease management interventions (0) | | **Existing**  **/implemented interventions** |
| --- | --- | --- |
| Organizational support | Integrated financing | **√** |
| Organizational support | Specific policies and subsidies for foreign population | - |
| Organizational support | Sustainable financing agreements with health insurers | **√** |
| Community | Communication platform | - |
| Community | Health market | - |
| Community | Cooperation with external community partners | **√** |
| Community | Multidisciplinary and transmural collaboration | **√** |
| Community | Role model in the area | - |
| Community | Regional collaboration for spread of the DMP | - |
| Community | Treatment and care pathways in out- and inpatient care | **√** |
| Community | Involvement of patient groups and/or panels in care design | - |
| Community | Regional training course | **√** |
| Community | Family participation | - |
| Self management | Promotion of disease specific information | - |
| Self management | Individual care plan | - |
| Self management | Life-style interventions (physical activity, diet, quit smoking) | **√** |
| Self management | Support of self-management (e.g. email or sms, e-consult) | - |
| Self management | Tele-monitoring | - |
| Self management | Personal coaching | - |
| Self management | Motivational interviewing | **√** |
| Self management | Informational meetings | - |
| Self management | Diagnosis and treatment of mental health issues | - |
| Self management | Reflection meetings | - |
| Self management | Group sessions for patients and family | - |
| Self management | Cognitive behavioural therapy | - |
| Decision Support | Care standards / Clinical guidelines | **√** |
| Decision Support | Uniform treatment protocol in outpatient and inpatient care | - |
| Decision Support | Training and independence of practise assistants | **√** |
| Decision Support | Professional education and training for care providers | **√** |
| Decision Support | Automatic measurement of process/outcome indicators | **√** |
| Decision Support | Care protocols for immigrants | - |
| Decision Support | Audit and feedback | **√** |
| Decision Support | Periodic evaluation of interventions and goal achievement | **√** |
| Decision Support | Structural participation in knowledge exchange | **√** |
| Decision Support | Quality of Life questionnaire | **√** |
| Decision Support | Qualitative evaluation of care via focus-groups with patients | - |
| Decision Support | Measurement of patient satisfaction | - |
| Delivery System Design | Delegation of care from specialist to nurse/care practitioner | **√** |
| Delivery System Design | Substitution of inpatient with outpatient care | **√** |
| Delivery System Design | Systematic follow-up of patients | - |
| Delivery System Design | One-stop outpatient clinic | - |
| Delivery System Design | Specific plan for immigrant population | - |
| Delivery System Design | Expansion of chain care to the secondary care setting | **√** |
| Delivery System Design | Joint consultation hours | - |
| Delivery System Design | Meetings of different disciplines for exchanging information | **√** |
| Delivery System Design | Monitoring of high-risk patients | **√** |
| Delivery System Design | Board of clients | - |
| Delivery System Design | Periodic discussions between professionals (and patients) | **√** |
| Delivery System Design | Stepped care method | - |
| ICT | Electronic Patient Records system with Patient Portal | - |
| ICT | Hospital or Practice Information System | **√** |
| ICT | Integrated Chain Information System | **√** |
| ICT | Use of ICT for Internal and/or regional benchmarking | **√** |
| ICT | Create a safe environment for data exchange | **√** |
| ICT | Systematic registration by every caregiver | **√** |
| ICT | Exchange of information between different care disciplines | **√** |

| **COPD disease management program: Archiatros / low quality of care***  * Based on the criteria of scoring programs using at least 34 interventions and implementing interventions within all six CCM dimensions as high-quality of care (1) versus those programs that implemented fewer disease management interventions (0) | | **Existing**  **/implemented interventions** |
| --- | --- | --- |
| Organizational support | Integrated financing | **√** |
| Organizational support | Specific policies and subsidies for foreign population | - |
| Organizational support | Sustainable financing agreements with health insurers | **√** |
| Community | Communication platform | - |
| Community | Health market | - |
| Community | Cooperation with external community partners | **√** |
| Community | Multidisciplinary and transmural collaboration | **√** |
| Community | Role model in the area | **√** |
| Community | Regional collaboration for spread of the DMP | - |
| Community | Treatment and care pathways in out- and inpatient care | **√** |
| Community | Involvement of patient groups and/or panels in care design | **√** |
| Community | Regional training course | **√** |
| Community | Family participation | - |
| Self management | Promotion of disease specific information | **√** |
| Self management | Individual care plan | **√** |
| Self management | Life-style interventions (physical activity, diet, quit smoking) | **√** |
| Self management | Support of self-management (e.g. email or sms, e-consult) | **√** |
| Self management | Tele-monitoring | - |
| Self management | Personal coaching | **√** |
| Self management | Motivational interviewing | **√** |
| Self management | Informational meetings | - |
| Self management | Diagnosis and treatment of mental health issues | - |
| Self management | Reflection meetings | - |
| Self management | Group sessions for patients and family | - |
| Self management | Cognitive behavioural therapy | - |
| Decision Support | Care standards / Clinical guidelines | **√** |
| Decision Support | Uniform treatment protocol in outpatient and inpatient care | - |
| Decision Support | Training and independence of practise assistants | **√** |
| Decision Support | Professional education and training for care providers | **√** |
| Decision Support | Automatic measurement of process/outcome indicators | **√** |
| Decision Support | Care protocols for immigrants | - |
| Decision Support | Audit and feedback | **√** |
| Decision Support | Periodic evaluation of interventions and goal achievement | - |
| Decision Support | Structural participation in knowledge exchange | **√** |
| Decision Support | Quality of Life questionnaire | **√** |
| Decision Support | Qualitative evaluation of care via focus-groups with patients | - |
| Decision Support | Measurement of patient satisfaction | - |
| Delivery System Design | Delegation of care from specialist to nurse/care practitioner | **√** |
| Delivery System Design | Substitution of inpatient with outpatient care | **√** |
| Delivery System Design | Systematic follow-up of patients | **√** |
| Delivery System Design | One-stop outpatient clinic | - |
| Delivery System Design | Specific plan for immigrant population | - |
| Delivery System Design | Expansion of chain care to the secondary care setting | - |
| Delivery System Design | Joint consultation hours | - |
| Delivery System Design | Meetings of different disciplines for exchanging information | **√** |
| Delivery System Design | Monitoring of high-risk patients | **√** |
| Delivery System Design | Board of clients | - |
| Delivery System Design | Periodic discussions between professionals (and patients) | **√** |
| Delivery System Design | Stepped care method | - |
| ICT | Electronic Patient Records system with Patient Portal | - |
| ICT | Hospital or Practice Information System | **√** |
| ICT | Integrated Chain Information System | - |
| ICT | Use of ICT for Internal and/or regional benchmarking | **√** |
| ICT | Create a safe environment for data exchange | - |
| ICT | Systematic registration by every caregiver | **√** |
| ICT | Exchange of information between different care disciplines | - |

| **COPD disease management program: Stichting Gezond Monnickendam / low quality of care***  * Based on the criteria of scoring programs using at least 34 interventions and implementing interventions within all six CCM dimensions as high-quality of care (1) versus those programs that implemented fewer disease management interventions (0) | | **Existing**  **/implemented interventions** |
| --- | --- | --- |
| Organizational support | Integrated financing | - |
| Organizational support | Specific policies and subsidies for foreign population | - |
| Organizational support | Sustainable financing agreements with health insurers | **√** |
| Community | Communication platform | - |
| Community | Health market | - |
| Community | Cooperation with external community partners | **√** |
| Community | Multidisciplinary and transmural collaboration | **√** |
| Community | Role model in the area | **√** |
| Community | Regional collaboration for spread of the DMP | **√** |
| Community | Treatment and care pathways in out- and inpatient care | **√** |
| Community | Involvement of patient groups and/or panels in care design | **√** |
| Community | Regional training course | **√** |
| Community | Family participation | - |
| Self management | Promotion of disease specific information | **√** |
| Self management | Individual care plan | **√** |
| Self management | Life-style interventions (physical activity, diet, quit smoking) | **√** |
| Self management | Support of self-management (e.g. email or sms, e-consult) | - |
| Self management | Tele-monitoring | - |
| Self management | Personal coaching | - |
| Self management | Motivational interviewing | **√** |
| Self management | Informational meetings | **√** |
| Self management | Diagnosis and treatment of mental health issues | **√** |
| Self management | Reflection meetings | - |
| Self management | Group sessions for patients and family | **√** |
| Self management | Cognitive behavioural therapy | - |
| Decision Support | Care standards / Clinical guidelines | **√** |
| Decision Support | Uniform treatment protocol in outpatient and inpatient care | - |
| Decision Support | Training and independence of practise assistants | **√** |
| Decision Support | Professional education and training for care providers | **√** |
| Decision Support | Automatic measurement of process/outcome indicators | **√** |
| Decision Support | Care protocols for immigrants | - |
| Decision Support | Audit and feedback | **√** |
| Decision Support | Periodic evaluation of interventions and goal achievement | - |
| Decision Support | Structural participation in knowledge exchange | **√** |
| Decision Support | Quality of Life questionnaire | **√** |
| Decision Support | Qualitative evaluation of care via focus-groups with patients | - |
| Decision Support | Measurement of patient satisfaction | - |
| Delivery System Design | Delegation of care from specialist to nurse/care practitioner | **√** |
| Delivery System Design | Substitution of inpatient with outpatient care | **√** |
| Delivery System Design | Systematic follow-up of patients | **√** |
| Delivery System Design | One-stop outpatient clinic | - |
| Delivery System Design | Specific plan for immigrant population | - |
| Delivery System Design | Expansion of chain care to the secondary care setting | - |
| Delivery System Design | Joint consultation hours | - |
| Delivery System Design | Meetings of different disciplines for exchanging information | **√** |
| Delivery System Design | Monitoring of high-risk patients | **√** |
| Delivery System Design | Board of clients | - |
| Delivery System Design | Periodic discussions between professionals (and patients) | **√** |
| Delivery System Design | Stepped care method | - |
| ICT | Electronic Patient Records system with Patient Portal | - |
| ICT | Hospital or Practice Information System | **√** |
| ICT | Integrated Chain Information System | - |
| ICT | Use of ICT for Internal and/or regional benchmarking | - |
| ICT | Create a safe environment for data exchange | - |
| ICT | Systematic registration by every caregiver | **√** |
| ICT | Exchange of information between different care disciplines | **√** |

| **COPD disease management program: Zorggroep Almere / high quality of care***  * Based on the criteria of scoring programs using at least 34 interventions and implementing interventions within all six CCM dimensions as high-quality of care (1) versus those programs that implemented fewer disease management interventions (0) | | **Existing**  **/implemented interventions** |
| --- | --- | --- |
| Organizational support | Integrated financing | **√** |
| Organizational support | Specific policies and subsidies for foreign population | **√** |
| Organizational support | Sustainable financing agreements with health insurers | **√** |
| Community | Communication platform | - |
| Community | Health market | - |
| Community | Cooperation with external community partners | **√** |
| Community | Multidisciplinary and transmural collaboration | **√** |
| Community | Role model in the area | - |
| Community | Regional collaboration for spread of the DMP | - |
| Community | Treatment and care pathways in out- and inpatient care | **√** |
| Community | Involvement of patient groups and/or panels in care design | **√** |
| Community | Regional training course | **√** |
| Community | Family participation | **√** |
| Self management | Promotion of disease specific information | **√** |
| Self management | Individual care plan | **√** |
| Self management | Life-style interventions (physical activity, diet, quit smoking) | **√** |
| Self management | Support of self-management (e.g. email or sms, e-consult) | - |
| Self management | Tele-monitoring | - |
| Self management | Personal coaching | **√** |
| Self management | Motivational interviewing | **√** |
| Self management | Informational meetings | **√** |
| Self management | Diagnosis and treatment of mental health issues | **√** |
| Self management | Reflection meetings | - |
| Self management | Group sessions for patients and family | **√** |
| Self management | Cognitive behavioural therapy | - |
| Decision Support | Care standards / Clinical guidelines | **√** |
| Decision Support | Uniform treatment protocol in outpatient and inpatient care | **√** |
| Decision Support | Training and independence of practise assistants | **√** |
| Decision Support | Professional education and training for care providers | **√** |
| Decision Support | Automatic measurement of process/outcome indicators | **√** |
| Decision Support | Care protocols for immigrants | - |
| Decision Support | Audit and feedback | **√** |
| Decision Support | Periodic evaluation of interventions and goal achievement | **√** |
| Decision Support | Structural participation in knowledge exchange | **√** |
| Decision Support | Quality of Life questionnaire | **√** |
| Decision Support | Qualitative evaluation of care via focus-groups with patients | **√** |
| Decision Support | Measurement of patient satisfaction | **√** |
| Delivery System Design | Delegation of care from specialist to nurse/care practitioner | **√** |
| Delivery System Design | Substitution of inpatient with outpatient care | **√** |
| Delivery System Design | Systematic follow-up of patients | **√** |
| Delivery System Design | One-stop outpatient clinic | - |
| Delivery System Design | Specific plan for immigrant population | - |
| Delivery System Design | Expansion of chain care to the secondary care setting | - |
| Delivery System Design | Joint consultation hours | - |
| Delivery System Design | Meetings of different disciplines for exchanging information | **√** |
| Delivery System Design | Monitoring of high-risk patients | **√** |
| Delivery System Design | Board of clients | **√** |
| Delivery System Design | Periodic discussions between professionals (and patients) | **√** |
| Delivery System Design | Stepped care method | - |
| ICT | Electronic Patient Records system with Patient Portal | - |
| ICT | Hospital or Practice Information System | **√** |
| ICT | Integrated Chain Information System | - |
| ICT | Use of ICT for Internal and/or regional benchmarking | **√** |
| ICT | Create a safe environment for data exchange | **√** |
| ICT | Systematic registration by every caregiver | - |
| ICT | Exchange of information between different care disciplines | **√** |

| **Diabetes disease management program: Huisartsen Coöperatie Zeist / high quality of care***  * Based on the criteria of scoring programs using at least 34 interventions and implementing interventions within all six CCM dimensions as high-quality of care (1) versus those programs that implemented fewer disease management interventions (0) | | **Existing**  **/implemented interventions** |
| --- | --- | --- |
| Organizational support | Integrated financing | **√** |
| Organizational support | Specific policies and subsidies for foreign population | - |
| Organizational support | Sustainable financing agreements with health insurers | - |
| Community | Communication platform | **√** |
| Community | Health market | - |
| Community | Cooperation with external community partners | **√** |
| Community | Multidisciplinary and transmural collaboration | **√** |
| Community | Role model in the area | **√** |
| Community | Regional collaboration for spread of the DMP | **√** |
| Community | Treatment and care pathways in out- and inpatient care | **√** |
| Community | Involvement of patient groups and/or panels in care design | **√** |
| Community | Regional training course | - |
| Community | Family participation | - |
| Self management | Promotion of disease specific information | **√** |
| Self management | Individual care plan | **√** |
| Self management | Life-style interventions (physical activity, diet, quit smoking) | **√** |
| Self management | Support of self-management (e.g. email or sms, e-consult) | - |
| Self management | Tele-monitoring | - |
| Self management | Personal coaching | **√** |
| Self management | Motivational interviewing | **√** |
| Self management | Informational meetings | - |
| Self management | Diagnosis and treatment of mental health issues | **√** |
| Self management | Reflection meetings | - |
| Self management | Group sessions for patients and family | **√** |
| Self management | Cognitive behavioural therapy | - |
| Decision Support | Care standards / Clinical guidelines | **√** |
| Decision Support | Uniform treatment protocol in outpatient and inpatient care | **√** |
| Decision Support | Training and independence of practise assistants | **√** |
| Decision Support | Professional education and training for care providers | **√** |
| Decision Support | Automatic measurement of process/outcome indicators | **√** |
| Decision Support | Care protocols for immigrants | - |
| Decision Support | Audit and feedback | **√** |
| Decision Support | Periodic evaluation of interventions and goal achievement | **√** |
| Decision Support | Structural participation in knowledge exchange | **√** |
| Decision Support | Quality of Life questionnaire | **√** |
| Decision Support | Qualitative evaluation of care via focus-groups with patients | - |
| Decision Support | Measurement of patient satisfaction | **√** |
| Delivery System Design | Delegation of care from specialist to nurse/care practitioner | **√** |
| Delivery System Design | Substitution of inpatient with outpatient care | **√** |
| Delivery System Design | Systematic follow-up of patients | **√** |
| Delivery System Design | One-stop outpatient clinic | - |
| Delivery System Design | Specific plan for immigrant population | - |
| Delivery System Design | Expansion of chain care to the secondary care setting | **√** |
| Delivery System Design | Joint consultation hours | - |
| Delivery System Design | Meetings of different disciplines for exchanging information | **√** |
| Delivery System Design | Monitoring of high-risk patients | - |
| Delivery System Design | Board of clients | - |
| Delivery System Design | Periodic discussions between professionals (and patients) | **√** |
| Delivery System Design | Stepped care method | **√** |
| ICT | Electronic Patient Records system with Patient Portal | - |
| ICT | Hospital or Practice Information System | **√** |
| ICT | Integrated Chain Information System | **√** |
| ICT | Use of ICT for Internal and/or regional benchmarking | **√** |
| ICT | Create a safe environment for data exchange | **√** |
| ICT | Systematic registration by every caregiver | **√** |
| ICT | Exchange of information between different care disciplines | **√** |

| **Diabetes disease management program: Zorggroep Haaglanden / low quality of care***  * Based on the criteria of scoring programs using at least 34 interventions and implementing interventions within all six CCM dimensions as high-quality of care (1) versus those programs that implemented fewer disease management interventions (0) | | **Existing**  **/implemented interventions** |
| --- | --- | --- |
| Organizational support | Integrated financing | **√** |
| Organizational support | Specific policies and subsidies for foreign population | **√** |
| Organizational support | Sustainable financing agreements with health insurers | **√** |
| Community | Communication platform | - |
| Community | Health market | - |
| Community | Cooperation with external community partners | **√** |
| Community | Multidisciplinary and transmural collaboration | - |
| Community | Role model in the area | - |
| Community | Regional collaboration for spread of the DMP | **√** |
| Community | Treatment and care pathways in out- and inpatient care | - |
| Community | Involvement of patient groups and/or panels in care design | **√** |
| Community | Regional training course | **√** |
| Community | Family participation | - |
| Self management | Promotion of disease specific information | **√** |
| Self management | Individual care plan | - |
| Self management | Life-style interventions (physical activity, diet, quit smoking) | - |
| Self management | Support of self-management (e.g. email or sms, e-consult) | - |
| Self management | Tele-monitoring | - |
| Self management | Personal coaching | **√** |
| Self management | Motivational interviewing | - |
| Self management | Informational meetings | - |
| Self management | Diagnosis and treatment of mental health issues | - |
| Self management | Reflection meetings | - |
| Self management | Group sessions for patients and family | - |
| Self management | Cognitive behavioural therapy | - |
| Decision Support | Care standards / Clinical guidelines | **√** |
| Decision Support | Uniform treatment protocol in outpatient and inpatient care | **√** |
| Decision Support | Training and independence of practise assistants | **√** |
| Decision Support | Professional education and training for care providers | **√** |
| Decision Support | Automatic measurement of process/outcome indicators | **√** |
| Decision Support | Care protocols for immigrants | - |
| Decision Support | Audit and feedback | - |
| Decision Support | Periodic evaluation of interventions and goal achievement | - |
| Decision Support | Structural participation in knowledge exchange | **√** |
| Decision Support | Quality of Life questionnaire | - |
| Decision Support | Qualitative evaluation of care via focus-groups with patients | - |
| Decision Support | Measurement of patient satisfaction | - |
| Delivery System Design | Delegation of care from specialist to nurse/care practitioner | - |
| Delivery System Design | Substitution of inpatient with outpatient care | **√** |
| Delivery System Design | Systematic follow-up of patients | - |
| Delivery System Design | One-stop outpatient clinic | - |
| Delivery System Design | Specific plan for immigrant population | - |
| Delivery System Design | Expansion of chain care to the secondary care setting | - |
| Delivery System Design | Joint consultation hours | - |
| Delivery System Design | Meetings of different disciplines for exchanging information | - |
| Delivery System Design | Monitoring of high-risk patients | - |
| Delivery System Design | Board of clients | - |
| Delivery System Design | Periodic discussions between professionals (and patients) | - |
| Delivery System Design | Stepped care method | **√** |
| ICT | Electronic Patient Records system with Patient Portal | - |
| ICT | Hospital or Practice Information System | **√** |
| ICT | Integrated Chain Information System | - |
| ICT | Use of ICT for Internal and/or regional benchmarking | **√** |
| ICT | Create a safe environment for data exchange | - |
| ICT | Systematic registration by every caregiver | **√** |
| ICT | Exchange of information between different care disciplines | - |

| **Diabetes disease management program: Gezondheidscentrum De Roerdomp / high quality of care***  * Based on the criteria of scoring programs using at least 34 interventions and implementing interventions within all six CCM dimensions as high-quality of care (1) versus those programs that implemented fewer disease management interventions (0) | | **Existing**  **/implemented interventions** |
| --- | --- | --- |
| Organizational support | Integrated financing | **√** |
| Organizational support | Specific policies and subsidies for foreign population | - |
| Organizational support | Sustainable financing agreements with health insurers | **√** |
| Community | Communication platform | - |
| Community | Health market | - |
| Community | Cooperation with external community partners | **√** |
| Community | Multidisciplinary and transmural collaboration | **√** |
| Community | Role model in the area | - |
| Community | Regional collaboration for spread of the DMP | - |
| Community | Treatment and care pathways in out- and inpatient care | **√** |
| Community | Involvement of patient groups and/or panels in care design | **√** |
| Community | Regional training course | **√** |
| Community | Family participation | - |
| Self management | Promotion of disease specific information | **√** |
| Self management | Individual care plan | - |
| Self management | Life-style interventions (physical activity, diet, quit smoking) | **√** |
| Self management | Support of self-management (e.g. email or sms, e-consult) | - |
| Self management | Tele-monitoring | - |
| Self management | Personal coaching | **√** |
| Self management | Motivational interviewing | **√** |
| Self management | Informational meetings | **√** |
| Self management | Diagnosis and treatment of mental health issues | - |
| Self management | Reflection meetings | - |
| Self management | Group sessions for patients and family | **√** |
| Self management | Cognitive behavioural therapy | - |
| Decision Support | Care standards / Clinical guidelines | **√** |
| Decision Support | Uniform treatment protocol in outpatient and inpatient care | **√** |
| Decision Support | Training and independence of practise assistants | **√** |
| Decision Support | Professional education and training for care providers | **√** |
| Decision Support | Automatic measurement of process/outcome indicators | **√** |
| Decision Support | Care protocols for immigrants | - |
| Decision Support | Audit and feedback | - |
| Decision Support | Periodic evaluation of interventions and goal achievement | - |
| Decision Support | Structural participation in knowledge exchange | **√** |
| Decision Support | Quality of Life questionnaire | - |
| Decision Support | Qualitative evaluation of care via focus-groups with patients | **√** |
| Decision Support | Measurement of patient satisfaction | **√** |
| Delivery System Design | Delegation of care from specialist to nurse/care practitioner | **√** |
| Delivery System Design | Substitution of inpatient with outpatient care | **√** |
| Delivery System Design | Systematic follow-up of patients | **√** |
| Delivery System Design | One-stop outpatient clinic | **√** |
| Delivery System Design | Specific plan for immigrant population | - |
| Delivery System Design | Expansion of chain care to the secondary care setting | **√** |
| Delivery System Design | Joint consultation hours | - |
| Delivery System Design | Meetings of different disciplines for exchanging information | **√** |
| Delivery System Design | Monitoring of high-risk patients | **√** |
| Delivery System Design | Board of clients | **√** |
| Delivery System Design | Periodic discussions between professionals (and patients) | **√** |
| Delivery System Design | Stepped care method | - |
| ICT | Electronic Patient Records system with Patient Portal | - |
| ICT | Hospital or Practice Information System | **√** |
| ICT | Integrated Chain Information System | - |
| ICT | Use of ICT for Internal and/or regional benchmarking | **√** |
| ICT | Create a safe environment for data exchange | **√** |
| ICT | Systematic registration by every caregiver | **√** |
| ICT | Exchange of information between different care disciplines | - |

| **Comorbidity disease management program: Chronische Ketenzorg Land van Cuijk en Noord Limburg BV / low quality of care***  * Based on the criteria of scoring programs using at least 34 interventions and implementing interventions within all six CCM dimensions as high-quality of care (1) versus those programs that implemented fewer disease management interventions (0) | | **Existing**  **/implemented interventions** |
| --- | --- | --- |
| Organizational support | Integrated financing | **√** |
| Organizational support | Specific policies and subsidies for foreign population | - |
| Organizational support | Sustainable financing agreements with health insurers | - |
| Community | Communication platform | - |
| Community | Health market | - |
| Community | Cooperation with external community partners | **√** |
| Community | Multidisciplinary and transmural collaboration | **√** |
| Community | Role model in the area | **√** |
| Community | Regional collaboration for spread of the DMP | - |
| Community | Treatment and care pathways in out- and inpatient care | **√** |
| Community | Involvement of patient groups and/or panels in care design | - |
| Community | Regional training course | **√** |
| Community | Family participation | - |
| Self management | Promotion of disease specific information | - |
| Self management | Individual care plan | - |
| Self management | Life-style interventions (physical activity, diet, quit smoking) | **√** |
| Self management | Support of self-management (e.g. email or sms, e-consult) | - |
| Self management | Tele-monitoring | - |
| Self management | Personal coaching | **√** |
| Self management | Motivational interviewing | **√** |
| Self management | Informational meetings | **√** |
| Self management | Diagnosis and treatment of mental health issues | - |
| Self management | Reflection meetings | - |
| Self management | Group sessions for patients and family | - |
| Self management | Cognitive behavioural therapy | - |
| Decision Support | Care standards / Clinical guidelines | **√** |
| Decision Support | Uniform treatment protocol in outpatient and inpatient care | **√** |
| Decision Support | Training and independence of practise assistants | **√** |
| Decision Support | Professional education and training for care providers | **√** |
| Decision Support | Automatic measurement of process/outcome indicators | **√** |
| Decision Support | Care protocols for immigrants | - |
| Decision Support | Audit and feedback | **√** |
| Decision Support | Periodic evaluation of interventions and goal achievement | - |
| Decision Support | Structural participation in knowledge exchange | **√** |
| Decision Support | Quality of Life questionnaire | - |
| Decision Support | Qualitative evaluation of care via focus-groups with patients | **√** |
| Decision Support | Measurement of patient satisfaction | **√** |
| Delivery System Design | Delegation of care from specialist to nurse/care practitioner | **√** |
| Delivery System Design | Substitution of inpatient with outpatient care | **√** |
| Delivery System Design | Systematic follow-up of patients | **√** |
| Delivery System Design | One-stop outpatient clinic | - |
| Delivery System Design | Specific plan for immigrant population | - |
| Delivery System Design | Expansion of chain care to the secondary care setting | **√** |
| Delivery System Design | Joint consultation hours | - |
| Delivery System Design | Meetings of different disciplines for exchanging information | **√** |
| Delivery System Design | Monitoring of high-risk patients | - |
| Delivery System Design | Board of clients | - |
| Delivery System Design | Periodic discussions between professionals (and patients) | **√** |
| Delivery System Design | Stepped care method | **√** |
| ICT | Electronic Patient Records system with Patient Portal | - |
| ICT | Hospital or Practice Information System | **√** |
| ICT | Integrated Chain Information System | **√** |
| ICT | Use of ICT for Internal and/or regional benchmarking | **√** |
| ICT | Create a safe environment for data exchange | **√** |
| ICT | Systematic registration by every caregiver | **√** |
| ICT | Exchange of information between different care disciplines | **√** |
